# Pulmonary Consumption, Bright’s Disease and Diabetes Mellitus

**Published:** 1906-06

**Authors:** B. F. Felchl

**Affiliations:** Chicago, Ill.


For Texas Medical Journal.

### Pulmonary Consumption, Bright's Disease and Diabetes Mellitus.

BY B. F. FELCHL, M. D., CHICAGO, ILL.

Pulmonary consumption, Bright's disease, and diabetes mellitus *are three diseases which are perpetuated by malnutrition.* Impaired digestion, constipation or dysentery, which may alternate, impaired heart action; the heart contracting with so little force as to throw but a small volume of blood to the extremities; hence cold feet and hands, weak nerves, insomnia, hysteria, irritable temper, torpid liver, tired feeling, dislike for manual exercise, but little muscular endurance, and pale, sallow, or what is called a muddy complexion. The rheumatic or gouty consumptive is prone to cystitis and a general irritability of the urinary tract, causing frequent micturation, the same as subjects of Bright's disease and diabetes. There are other symptoms common to these three diseases.

DIABETES MELLITUS

is not an uncommon complication of pulmonary consumption, and the affection of the liver and kidneys frequently hastens the death of the patient. The life of the so-called consumptive with a muddy complexion is frequently in greater danger from disease of the liver than of the lungs. *Please don't give mercury in either of the foregoing diseases. It gives but temporary relief and almost invariably does harm in all uric acid diseases.*

BRIGHT'S DISEASE AND DIABETES MELLITUS

are uric acid diseases and pulmonary consumption may be. The first two diseases are frequently coupled with gout or rheumatism in the same individual, and sometimes albumen and sugar in the urine at the same time. At other times they may alternate. These two diseases are very similar, having twelve or fifteen symptoms in common, and four to seven on which we rely to distinguish one from the other.

ALL CONSUMPTIVES

may be injured by the retention of uric acid, as defective lungs cause the production of an excess of urates for want of proper oxygenation of the blood. To better the condition of patients suffering from pulmonary consumption, Bright's disease or diabetes mellitus, *digestion is of the first importance.* Much depends upon diet concerning which no iron rule should be made, but must vary with different patients and with different conditions of the same patient. What digests readily with one may not with another under the same or similar conditions. Cake and pastry in general, and too much sweets in any form, must be avoided. Meat, as a general rule, should not be tasted to exceed once per day and frequently should be prohibited, as it tends to produce constipation, acidity and uric acid. Fat meat and bacon, when it agrees with the patient, are beneficial. It may be fried crisp or boiled. Of meat, perhaps chicken or turkey and game are the easiest to digest. Fresh fish is excellent. Avoid meat extracts and beef tea. They are mere stimulants from the xanthin they contain, which is practically uric acid. Of vegetables, especially *beans, tomatoes, and rhubarb, should be prohibited.* The diabetic should also usually deny himself potatoes. People with either of the above diseases are injured more than benefited by acid fruit. Tea and coffee injure digestion, the nerves, the heart, cause constipation and insomnia, besides being one of the most direct methods of filling a person with uric acid, thereby perpetuating and intensifying these diseases. *Strong ale and beer* and sweet wine are sometimes worse than tea and coffee. Dilute whiskey, when taken with moderation, if it does not injure the stomach or kidneys, is quite beneficial. It is generally conceded that milk, when it agrees with a patient, is the best article of diet, yet for the diabetic it is not always desirable, as it tends to diuresis. Buttermilk is generally beneficial. *Regular outdoor exercise each day is an essential and almost an indispensable requisite for any permanent improvement in either of the above diseases.* When the patient is put to bed, he journeys toward the undertaker. Walking each day as far as possible without overexertion is perhaps the best exercise for the consumptive. Too much work is injurious. *Muscular strength must be built up and circulation developed as fast as possible.* To this end the patient should be massaged once per day when he can afford it; otherwise not less than twice per week. Consumptives whose circulation in the axillary regions is generally seriously impeded *most especially require massage.* Medicine should be given to assist digestion, to correct the bowels and urinary irritation, to excrete urates, to build up the heart, the nervous system, and to correct any other ailment of each individual patient. To prevent injury to the heart and to digestion, drugs to reduce acidity and drugs to excrete urates should be administered in full doses, and in chronic cases but once per day and not to exceed four days per week. In Bright's disease, as a rule, opiates should not be given; in diabetes mellitus but seldom. We sometimes give Dover powders at bedtime. In pulmonary consumption in certain stages we don't know how to get along without Dover powders, and our latest and best cough syrup contains more or less codein or heroin. The special ailment of each patient must be looked after without much regard to the general disease for which the patient is being treated. People with either of the three diseases above specified, treated in accordance with the above suggestions, will make almost immediate improvement. *These are not fatal diseases when properly treated.* And *unless one or more*

*of their vital organs are injured beyond possible repair, their nutrition will continue to improve until they are happy.*

The diabetics above referred to are the true diabetics, with diminished combustion and temperature and more or less emaciated.

The so-called diabetics, who excrete sugar in their urine and are fat and ruddy, lack the essential symptoms of diabetes, and must be treated for obesity; their diet and beverages gradually reduced, their exercise increased, and their extra flesh rubbed off. They are not diabetics; they eat and drink too much and the escape of the sugar which, with them, is surplus alimentation, may have saved them from gout or apoplexy.

The common ailment known as old age, which is usually observed at fifty and is conspicuous at sixty, may affect mankind at any period after thirty-five years to their journey's end. This ailment may properly be classified with the three above diseases, the health and strength of the old rebuilt and their lives extended by the treatment above suggested.

